# Moving beyond SUV_max_: volumetric and heterogeneous dose evaluation in ^18^F-BPA PET-guided BNCT

**DOI:** 10.1007/s11604-026-02047-w

**Published:** 2026-07-01

**Authors:** Yuan-Hao Liu

**Affiliations:** 1 Neuboron Medtech Ltd, Bazhong, Sichuan China; 2 Xiamen Humanity Hospital, Xiamen, Fujian China

**Keywords:** BNCT, F-BPA, Heterogeneous uptake, TNR

## Dear Editor

I read with great interest the article by Yoshino et al. [1], which evaluated whether tumor maximum standardized uptake value (SUV_max_) from ^18^F-BPA PET and SUV_max_-derived BNCT tumor dose correlate with complete response in head and neck cancer. The authors reported that neither SUV_max_ nor the conventional individual-model dose parameters significantly predicted complete response. I consider this finding important and credible; however, its implication should be interpreted with caution. The result does not suggest that ^18^F-BPA PET lacks clinical value. Rather, it highlights the limitation of reducing a spatially heterogeneous theragnostic signal to a single-voxel maximum value. In other words, intratumoral heterogeneity of boron distribution is a crucial determinant of BNCT dose delivery and treatment response, and should be carefully evaluated using the full volumetric information provided by ^18^F-BPA PET, rather than relying solely on SUV_max_.

BNCT efficacy is largely governed by the boron dose component, which depends on the spatial coincidence of boron concentration, thermal neutron fluence, and biological effectiveness, as illustrated in Fig. 1. SUV_max_ represents only the hottest voxel or small subregion within the tumor. It does not describe intratumoral cold spots, necrosis, heterogeneous perfusion, viable tumor burden, or the relationship between boron uptake and neutron fluence. Therefore, deriving a tumor-to-blood ratio from SUV_max_ and applying it uniformly to the entire gross tumor volume is biologically and dosimetrically questionable. This approach effectively assumes that the whole tumor receives the boron concentration of its most avid PET region, which may substantially overestimate dose coverage.

**Fig. 1:**
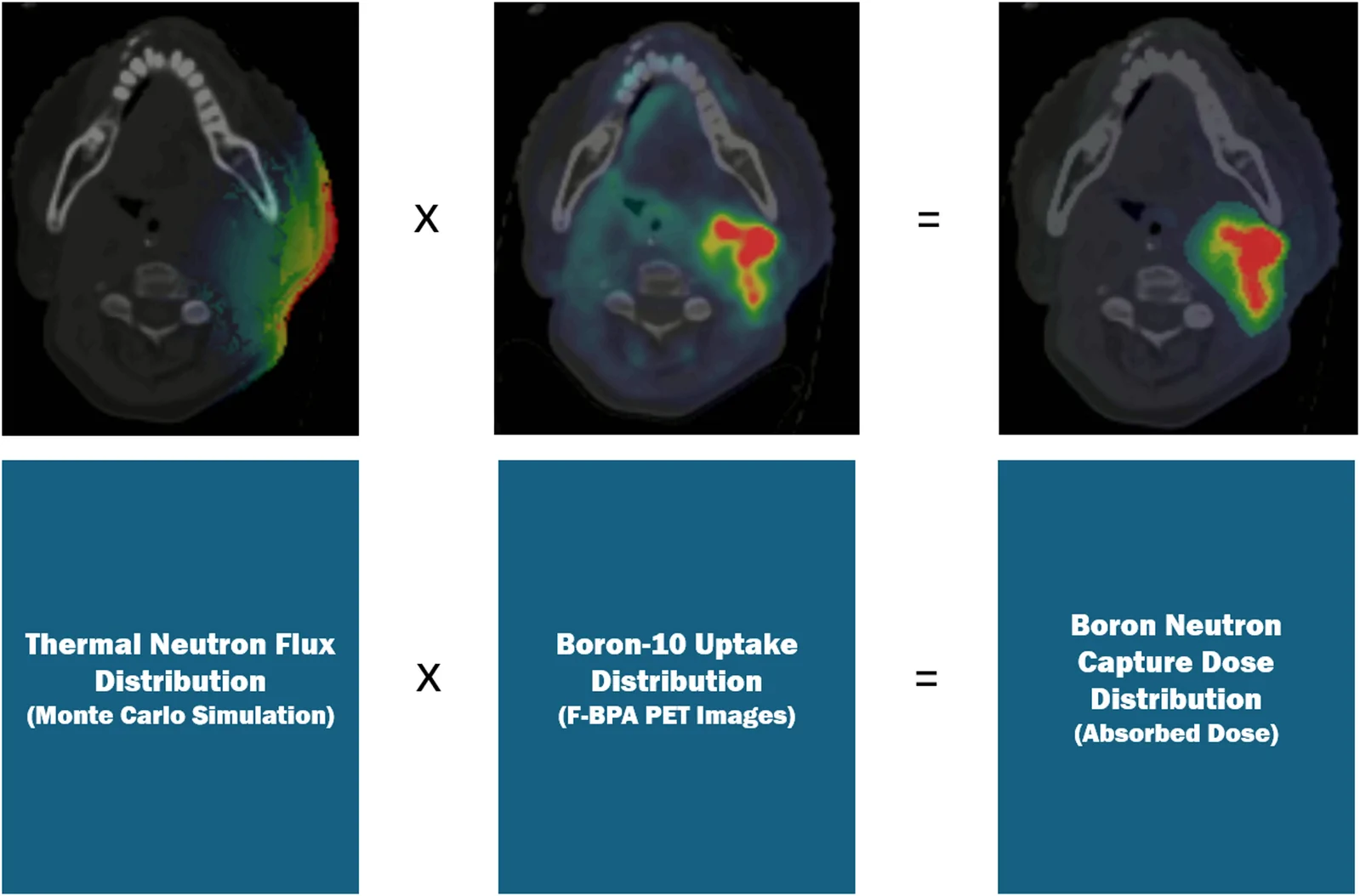
BNCT dose formation from neutron flux and boron uptake. Thermal neutron flux from Monte Carlo calculation and boron-10 uptake estimated by ^18^F-BPA PET jointly determine the resulting boron neutron capture dose distribution

Yoshino et al.’s results are consistent with this concern. The non-complete-response group showed numerically higher median SUV_max_ and SUV_max_-derived individual-model D_80_ than the complete-response group, although the differences were not statistically significant. Such a direction of effect is difficult to reconcile with SUV_max_ being a valid dose-response predictor. The key message, therefore, should not be that ^18^F-BPA PET failed, but that SUV_max_-based homogeneous individual modeling was insufficient as a response predictor.

This interpretation is supported by prior clinical evidence. Aihara et al. reported that, in recurrent head and neck squamous cell carcinoma treated with BNCT, T_min_/N rather than T_max_/N was the only ^18^F-BPA PET parameter significantly associated with complete response [2]. This is clinically intuitive: local tumor control is determined by the survival of clonogenic tumor cells, which are more likely to persist in low-boron or underdosed subvolumes than in regions showing maximum tracer uptake. Therefore, maximum uptake is intrinsically less relevant to tumor control than adequate dose coverage of the lowest-uptake viable tumor regions. In other words, SUV_max_ provides little information about dose cold spots, which are more clinically relevant to incomplete tumor regression and subsequent recurrence.

Recent dosimetric evidence further reinforce this point. Kobayashi et al. showed that incorporating heterogeneous ^18^F-BPA-derived boron distribution into dose evaluation produced significantly lower tumor dose indices than conventional uniform models or maximum-value-derived tumor-to-blood models [3].

At the same time, the physiologic rationale for ^18^F-BPA PET remains strong. Li et al. showed that BPA and ^18^F-BPA share broadly comparable transport and biodistribution characteristics, supporting ^18^F-BPA PET as a useful surrogate for estimating boron distribution, while also acknowledging biological and quantitative imperfections [4]. Thus, the appropriate response is not to discard ^18^F-BPA PET, but to use its volumetric information more rationally.

A more appropriate framework would integrate ^18^F-BPA PET into heterogeneous TPS-based dosimetry and evaluate the resulting dose-volume histogram. Near-minimum coverage indices such as D_95_ or D_98_, together with D_80_, D_mean_, and cold-volume metrics, are more biologically meaningful than SUV_max_ because they assess whether the biologically active tumor volume receives adequate dose. SUV_mean_ may be less noisy than SUV_max_ and may better reflect average boron uptake, but it still cannot identify clinically relevant cold subvolumes.

Future studies should prospectively evaluate PET-informed heterogeneous boron distribution, neutron fluence, and DVH-derived indices, while carefully addressing image registration, PET resolution, and tumor heterogeneity. PET-derived heterogeneous boron-distribution correction has already been shown to alter DVH patterns and reduce overestimation of low-dose tumor regions, supporting its incorporation into BNCT treatment planning [5].

In summary, Yoshino et al. have identified a vital clinical blind spot. Their findings should be viewed as evidence against SUV_max_-based homogeneous individual modeling, not against ^18^F-BPA PET-guided BNCT planning. The field should move from “maximum uptake” toward “adequate heterogeneous dose coverage of the whole biologically active tumor.”

## Data Availability

No datasets were generated or analyzed in this work.
